# Peer-mentorship and first-year inclusion: building belonging in higher education

**DOI:** 10.1186/s12909-023-04805-0

**Published:** 2023-11-07

**Authors:** Gisela J. van der Velden, John A.L. Meeuwsen, Christine M. Fox, Cecily Stolte, Gönül Dilaver

**Affiliations:** Education Center, University Medical Center Utrecht, Utrecht University, Heidelberglaan 100, HB 4.05, Utrecht, 3584 CX The Netherlands

**Keywords:** Authenticity, Belonging, Inclusive learning environment, Peer-mentorship, Transition to HE

## Abstract

**Background:**

An inclusive academic environment is pivotal to ensure student well-being and a strong sense of belonging and authenticity. Specific attention for an inclusive learning environment is particularly important during a student’s transition to higher education. At Utrecht University’s Medical School, explorative interviews with students from minority groups indicated they did not always feel included during the orientation programme of their academic education. We, therefore, developed a bias awareness training with theoretical and practical components on diversity and inclusion for peer-mentors who are assigned to each first-year student at the start of university.

**Methods:**

At the end of the orientation programme, we investigated the effectiveness of the training for two consecutive years using two measurements. Firstly, we investigated the behavioural changes in the peer-mentors through a (self-reporting) questionnaire. Additionally, we measured the perceived inclusion of the first-year students, divided into belonging and authenticity, using a validated questionnaire.

**Results:**

Our results show that peer-mentors found the training useful and indicated it enabled them to create an inclusive atmosphere. Overall, students experienced a high level of inclusion during the orientation programme. After the first year, the bias training was adjusted based on the evaluations. This had a positive effect, as mentors felt they were significantly more able to provide an inclusive orientation in the second year of this study. In line with this, students experienced an increased level of authenticity specifically due to the peer-mentor in the second year as compared to the first.

**Conclusions:**

We conclude that training peer-mentors is an effective way to increase awareness and to ensure an inclusive atmosphere during the start of higher education.

**Supplementary Information:**

The online version contains supplementary material available at 10.1186/s12909-023-04805-0.

## Background

The population of the Netherlands is becoming more diverse with an estimated 25% having a migrant background [1]. This diversity, however, is not translating into university classrooms. The reason for this is complex. Social-economic disadvantage and university admission requirements often make it difficult for those wishing to attend. Additionally, the lack of role models in higher education (HE) and in their surroundings, potentially discourages students with minority backgrounds from applying. While the statistics differ between universities, a 2015 study has shown that Utrecht University was the least diverse with only 22% of the student population having a migration background [2]. This study also showed that these students, who managed to overcome their disadvantages, were more likely to drop out of university during or after their first year [2]. These findings align with previous research on secondary schools, which showed that students with minority backgrounds were more at risk of leaving school before obtaining their degree [3]. While there are several factors for these statistics, one possible reason for this high dropout rate could be a weak sense of belonging.

A strong sense of belonging is an important factor for a student’s well-being and increases retention in higher education [4]. Diversity in the classroom has also been shown to benefit all students as it improves study and team performance [5–8]. Moreover, diversity in the classroom helps prepare students for societal challenges they will experience outside of university [9–13]. Therefore, to make diversity truly successful, specific focus on inclusion is essential.

### Inclusion

For all people it is important to feel included. It enhances self-esteem and it validates and strengthens beliefs and world views [7, 14]. Inclusion is also essential when it comes to students’ performances, their well-being, and mutual understanding and acceptance [7, 14].

For a group to be inclusive, all members must feel appreciated and heard [7, 15, 16]. This idea aligns with Shore et al. (2011) who defines inclusion as ‘the degree to which [a student] perceives that they are an esteemed member of the work group through experiencing treatment that satisfies their needs for *belongingness* and *uniqueness*’ [7]. Jansen et al. (2014) amended this definition by replacing *uniqueness* with *authenticity*, as described in the Self Determination Theory by Ryan and Deci (2000) [14, 17].

According to Jansen et al. (2014), there are three important features surrounding the notion of inclusion. Firstly, inclusion satisfies individual needs. Secondly, it is the group that includes the individual and not the individual who connects to the group. Finally, the concept of inclusion consists of two main components, *belonging* and *authenticity* [14]. While individuals must feel that they are part of the group, they must also feel that the group grants them enough space and encourages them to be their authentic selves, even if, in some ways, that makes them different from the rest. Belonging and authenticity must be in balance for an individual to experience optimal inclusion [7, 14, 18]. In line with this, an inclusive academic environment results in increased student well-being and a stronger sense of belonging and authenticity for all students [12]. It is the institution’s responsibility to create an educational environment where all students feel included in order to realize the positive effects of diversity in the transition to HE [10]. Therefore, a sense of belonging throughout a student’s academic career is of major importance [7, 11, 14], especially during the university’s orientation program where first-year students enter a new phase of their lives in HE.

### Support of inclusion through peer-mentoring

One way to help create inclusion within the educational environment is through peer-mentoring [19]. As Barack Obama said in 2012, ‘A supportive mentor can mean the difference between struggle and success’ [20]. A growing number of studies have shown that guidance through mentoring programs vastly increases performance and inclusion of students [5, 10, 21, 22]. Mentoring has also shown to lead to increased retention [23] and a higher success rate in the (academic) career of students [21]. Additionally, a handful of studies have also shown the effectiveness of peer-mentors and the role they can play for incoming students’ transition to HE [24–27].

Mentors can also inform and positively influence their mentees on topics related to diversity and inclusion, such as unconscious bias, self-awareness, and micro-aggressions [28]. They can also help students psychologically. Through informal interactions, students and their mentors can ‘establish close relationships that go beyond providing career guidance and technical skill training’ [29]. This comfort can increase the well-being and perceived inclusion amongst students. For a mentor to successfully connect with a mentee, they must become aware of the existence of their implicit bias. It has been shown that retention of students is highly influenced by the institutional culture and its unconscious social practices [30]. Many students who leave university in the first year, often do so due to environmental factors rather than intellectual problems [31]. An inclusive academic environment is therefore important to ensure a smooth transition into HE [32]. Previous studies have shown that bias awareness trainings can be effective, especially in a medical setting [33–37]. Therefore, to ease the transition for first year university students, mentors should be educated on their (unconscious) biases [21, 38].

### Purpose

At Utrecht University’s School of Medicine, students with diverse backgrounds, including religion, gender, and sexuality, come together. A 2014 study found that an estimated 12% of first-year students within the school were from minority backgrounds [2]. This study also showed that these students were more likely to drop out in or after their first year compared with their non-minority peers (14.5% vs. 12.5%) [2]. The challenge that the Utrecht School of Medicine faces is creating a feeling of inclusion and equal opportunity to help retain minority students, and therefore, reap the benefits of their inclusion [5–8].

### This study

The School of Medicine at Utrecht University offers two undergraduate programs, Biomedical Sciences, and Medicine. At the start of their first year, all students in the School of Medicine are assigned a peer-mentor who guides them through the orientation period. This orientation period includes social activities as well as academic guidance for the first few weeks of university. Our overarching research question for this study was to see how offering peer-mentors a bias awareness training impacts the perceived inclusion of first year students.

To answer this question, an explanatory multiphase mixed method study was conducted. The first part of this study was to add a bias awareness training to the pre-existing peer-mentorship program. Over the course of two years, we then investigated the self-reported effects of the training on the mentors and mentees. The findings from this study demonstrate the effect of bias awareness training of mentors and whether it can help increase the perceived inclusion in higher education to improve retention of students with minority backgrounds.

## Methods

### Study design

Prior to our investigation, focus-group discussions were held with students from culturally diverse backgrounds from the School of Medicine at Utrecht University. These students reported that, particularly during social activities, they felt less included, making their personal and social integration more difficult. At the School of Medicine, first-year Biomedical Sciences and Medicine students begin their academic year with an orientation program where they are assigned a peer-mentor. The orientation, which lasts roughly two months, consists of a variety of activities that facilitate student learning in the transition to HE as well as personal and social integration.

**Fig. 1:**
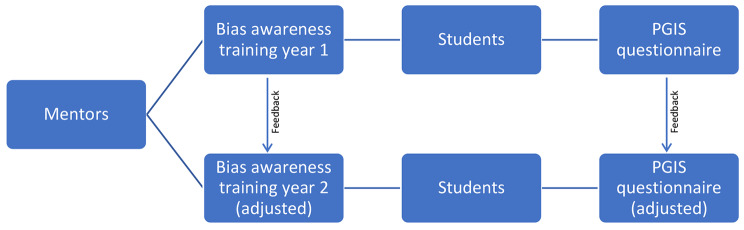
**Flow-chart of research study**. Feedback from participants after year 1 of the study was used to adjust the bias awareness training and survey and were implemented in year 2 of the study

As a way of addressing the comments made during the focus-group discussions, we conducted a two-year explanatory multiphase mixed method study where peer-mentors were first given a bias training before their mentorship. Peer-mentors were chosen for the intervention because they are often the first point of contact for incoming students at the School of Medicine and are often perceived as more approachable than faculty members. This study design allowed us to evaluate our findings and then implement them in the second year to help determine if our interventions had positive outcomes and should be applied long-term to achieve our goal of creating a more diverse and inclusive learning environment at the School of Medicine. A flow-chart of the study design can be found in Fig. 1.

#### Theoretical framework for bias awareness training

The bias awareness training we designed was based on a six-point framework published by Sukhera and Watling in 2018 [39]. While there are several different frameworks [40–43], the Sukhera and Watling framework was chosen because it was designed for integrating implicit bias recognition into the education curriculum of health professions [39]. The framework includes six key features: (1) creating a safe and nonthreatening learning context, (2) increasing knowledge about the science of implicit bias, (3) emphasizing how implicit bias influences behaviours and patient outcomes, (4) increasing self-awareness of existing implicit biases, (5) improving conscious efforts to overcome implicit bias, and (6) enhancing awareness of how implicit bias influences others [39]. A full description of the bias awareness training can be found in appendix D.

### Data collection

The study design and survey were approved by the Ethical Review Board of the Dutch Association for Medical Education (Nederlandse Vereniging voor Medisch Onderwijs; NVMO), dossier number 2018.6.12. Informed consent was obtained from all individual participants included in this study.

#### Participants

In each year of our study, 40 second- and third-year peer-mentors from the Biomedical Sciences and Medicine bachelors’ programmes (80 in total for two years), participated in the bias awareness training prior to the first-year orientation programme.

Additionally, each year a total of 479 first-year students are welcomed to the School of Medicine at Utrecht University: 175 students for Biomedical Sciences and 304 students for Medicine.

#### Sampling technique

At the end of the bias-awareness training programme, the peer-mentors were sent a survey (described below). They had one month to complete the survey and received a reminder after two weeks. The survey was distributed using Formdesk, all peer-mentors were invited to fill in the survey via e-mail, which also included an explanation of the study and an informed consent form.

At the end of the orientation programme, the first-year students received a survey (described in below) and were given a month to fill it in. The survey was distributed using Formdesk, all students were invited to fill in the survey via e-mail, which also included an explanation of the study and an informed consent form.

#### Sample size

Due to the small sample size of peer-mentors for each study year, the data for both years was combined. Overall, 31 peer-mentors completed the survey out of the 80 possible participants. The respondents included 14 Biomedical Sciences and 17 Medical students. Thirteen of the 31 respondents identified as male and 18 identified as female. Twenty of the peer-mentors were in the second year of their degree program and eleven in their third year. They were all first-time peer-mentors. The majority of the peer-mentors were born in the Netherlands and had parents who possessed a HE degree. Most indicated they identified as heterosexual and non-religious.

In the first year of our study, of the 479 first-year students who were asked to fill in the survey, 91 (19%) participated in the study. Forty-five respondents (49%) were Biomedical Sciences students and 50 (51%) were Medical students. Of all respondents, 25 identified as male and 69 identified as female. One respondent did not disclose their gender.

In the second year of our study, 112 first-year students participated in the study. Sixty-four respondents were Biomedical Sciences students, 47 were Medical students, one respondent did not disclose which degree programme they were following. Of all respondents, 28 identified as male and 83 identified as female.

#### Survey

The peer-mentor survey (Appendix A) consisted of three sections. The first section (Q1-Q10) was designed to gain an overview of the demographics of our peer-mentors, such as age, gender, and sexual orientation. The second section contained questions to measure the extent to which the peer-mentors felt that the training contributed to an increase in their knowledge and awareness about diversity and inclusion and whether they felt able to apply this knowledge to make first-year students feel more included. The third section contained the following open-ended questions: (1) Can you think of a situation or an activity during which you were able to implement the knowledge you gained from the bias training? (2) Can you think of a situation or an activity during which, due to the bias training, you made sure that specific students would feel accepted, welcome and at home? (3) Can you think of a situation or an activity during which, due to the bias training, you noticed that someone did not feel accepted, welcome or at home? (4) Can you think of an activity from the introduction programme which you would like to adapt or omit from the programme for next year because it was not inclusive for all students? (5) Do you have any additional comments or questions?

To collect the data from first-year students, an additional survey was designed (see Appendix B). The survey was divided into three sections. The first section (Q1-8) was designed to gain an overview of the demographics of our first-year students, such as age, gender, and sexual orientation. The second section continued with questions (Q9-40) from the Perceived Group Inclusion Scale (PGIS) [14] to measure the extent to which students felt included. The PGIS is a validated questionnaire comprising 16 questions to measure social inclusion [14]. It provides a measure for the extent to which someone feels part of the group (belonging) and can be their authentic selves within the group (authenticity). Students were asked to think to what extent both their mentor and their peers contributed to their sense of belonging and authenticity. These questions made use of a five-point Likert scale (from ‘totally agree’ to ‘totally disagree’). The third section of the survey comprised three qualitative open-ended questions: (1) In which way did the orientation committee and/or the mentor make sure you felt accepted, appreciated, and welcome or contributed to a high sense of belonging during the orientation period? (2) Which improvements can the orientation committee and/or mentor make during the orientation period to make sure you feel accepted, appreciated, and welcome or to contribute to a higher sense of belonging? (3) You can leave any additional comments here.

In the second year, the survey which was used in year 1 was slightly adjusted (Appendix C). This was based on feedback received from the students in the previous year who indicated that the PGIS section contained repetitive questions. For instance, within the questions about belonging there were questions about a more active and passive role of the mentor/peer. The same holds true for the questions about authenticity. From each category, two passive and two active questions were eliminated based on similarity with other questions in the survey. The questions removed from the first-year survey were numbers 10, 11, 13, 15, 17, 20, 21 and 24. This left 16 questions in the PGIS portion: eight about belonging and eight about authenticity (see appendix for the entire survey). The remaining sections of the survey were kept the same as year 1, albeit in a different order. See Table 1 below for an overview of the PGIS questions used in each year.

**Table 1:** Overview of PGIS questions used in 2018 survey and 2019 survey

PGIS 2018	PGIS 2019
During the introduction, the group of my fellow students…	During the introduction, the group of my fellow students…
Q 9: …gives me the feeling I belong to the group.	Q 9: …gives me the feeling I belong to the group.
Q 10: …gives me the feeling I am part of the group.	
Q 11: …gives me the feeling I fit in the group.	
Q 12: …treats me like an insider.	Q 11: …treats me like an insider.
Q 13: …likes me.	
Q 14: …values me.	Q 13: …values me.
Q 15: …is happy with me.	
Q 16: …cares for me.	Q 15: …cares for me.
Q 17: …allows me to be authentic.	
Q 18: …allows me to be who I am.	Q 10: …allows me to be who I am.
Q 19: …allows me to express my authentic self.	Q 12: …allows me to express my authentic self.
Q 20: …allows me to show myself as I am.	
Q 21: …encourages me to be authentic.	
Q 22: …encourages me to be who I am.	Q 14: …encourages me to be who I am.
Q 23: …encourages me to express my authentic self.	Q 16: …encourages me to express my authentic self.
Q 24: …encourages me to show myself as I am.	
During the introduction, the mentor…	During the introduction, the mentor…
Q 25: …gives me the feeling I belong to the group.	Q 17: …gives me the feeling I belong to the group.
Q 26: …gives me the feeling I am part of the group.	
Q 27: …gives me the feeling I fit in the group.	
Q 28: …treats me like an insider.	Q 19: …treats me like an insider.
Q 29: …likes me.	
Q 30: …values me.	Q 21: …values me.
Q 31: …is happy with me.	
Q 32: …cares for me.	Q 23: …cares for me.
Q 33: …allows me to be authentic.	
Q 34: …allows me to be who I am.	Q 18: …allows me to be who I am.
Q 35: …allows me to express my authentic self.	Q 20: …allows me to express my authentic self.
Q 36: …allows me to show myself as I am.	
Q 37: …encourages me to be authentic.	
Q 38: …encourages me to be who I am.	Q 22: …encourages me to be who I am.
Q 39: …encourages me to express my authentic self.	Q 24: …encourages me to express my authentic self.
Q 40: …encourages me to show myself as I am.	

### Data analysis

#### Quantitative analysis

Analysis of the questions for the peer-mentors using a Likert scale were performed with RStudio [44] and the R software package ( [45] version 3.5.1; R Foundation for Statistical Computing, Vienna, Austria). The scores of each question were calculated for the two years separately and for the total study. The Mann-Whitney U test was applied due to the abnormal distribution of the data. We considered a p-value < 0.05 as statistically significant.

The first-year students received the PGIS questionnaire. Although it is a validated questionnaire, we determined the internal consistency of the questions in our sample for the factors belonging and authenticity regarding mentors and peer students. The Internal consistency reliability, indicated by the Cronbach’s Alpha variable, was calculated using SPSS (IBM SPSS Statistics for Windows, version 26, IBM Corp., Armonk, N.Y., USA).

The answers to the PGIS-questions were then compared between the different indicated demographic variables in order to investigate whether the experienced inclusion differed per variable. These analyses were performed with RStudio [44] and the R software package ( [45] version 3.5.1; R Foundation for Statistical Computing, Vienna, Austria). Non-normal distributed data were then compared with the Mann-Whitney U test. We considered a p-value < 0.05 as statistically significant.

#### Qualitative analysis

The open-ended questions for the peer-mentors were analysed by two coders/researchers from the HE field, using the three steps as described by Dörnyei (2007): data reduction, data display, and data interpretation (Dörnyei, 2007, p245-253). The first three open-ended questions regarding knowledge-application were analysed together, resulting in six overarching themes, while the question about improvements to the training was treated separately, resulting in four themes. We reported the results based on the experts’ categorizations.

For data reduction, an in-depth analysis was performed. Due to the complexity of individual answers to open-ended questions, we created categories for similar responses. This was realized by first looking for common answers. For example, whenever the respondents used the same kind of words or expressed comparable ideas. For a better overview, we also highlighted similar answers in the same colour. Second, the answers with the same colours were assigned labels. The labels were short notes or sentences which summarized the content of the common responses. Dörnyei (2007) supports this approach by stating that “[t]here will inevitably be some similar or closely related [answers], which can be clustered together under a broader label” (p. 247). Accordingly, the broader labels were changed into shortened category names [47]. The number of responses in each category were then recorded.

This first step facilitated the presentation and discussion of the results in the second and third step of the content analysis approach (data display and data interpretation) which will be presented later in this paper.

The first two open-ended questions for the first-year students were analysed separately through a qualitative approach by forming categories as described above for the mentor survey [46, 47]. The results from 2018 to 2019 were merged as many of the issues and concerns overlapped.

## Results

Using an explanatory multiphase mixed method study, we investigated whether a bias awareness training would increase the awareness of implicit bias in peer-mentors for the orientation program at the Utrecht University School of Medicine. We also investigated whether the training would enable peer-mentors to provide a more inclusive orientation for beginning first-year students and whether this would lead to a higher feeling of inclusion and authenticity in first-year students. Below the results have been divided into two parts: ‘mentor’ and ‘student’ results. Each of these has been subdivided into quantitative and qualitative results.

### Effect of training on mentor awareness

**Fig. 2:**
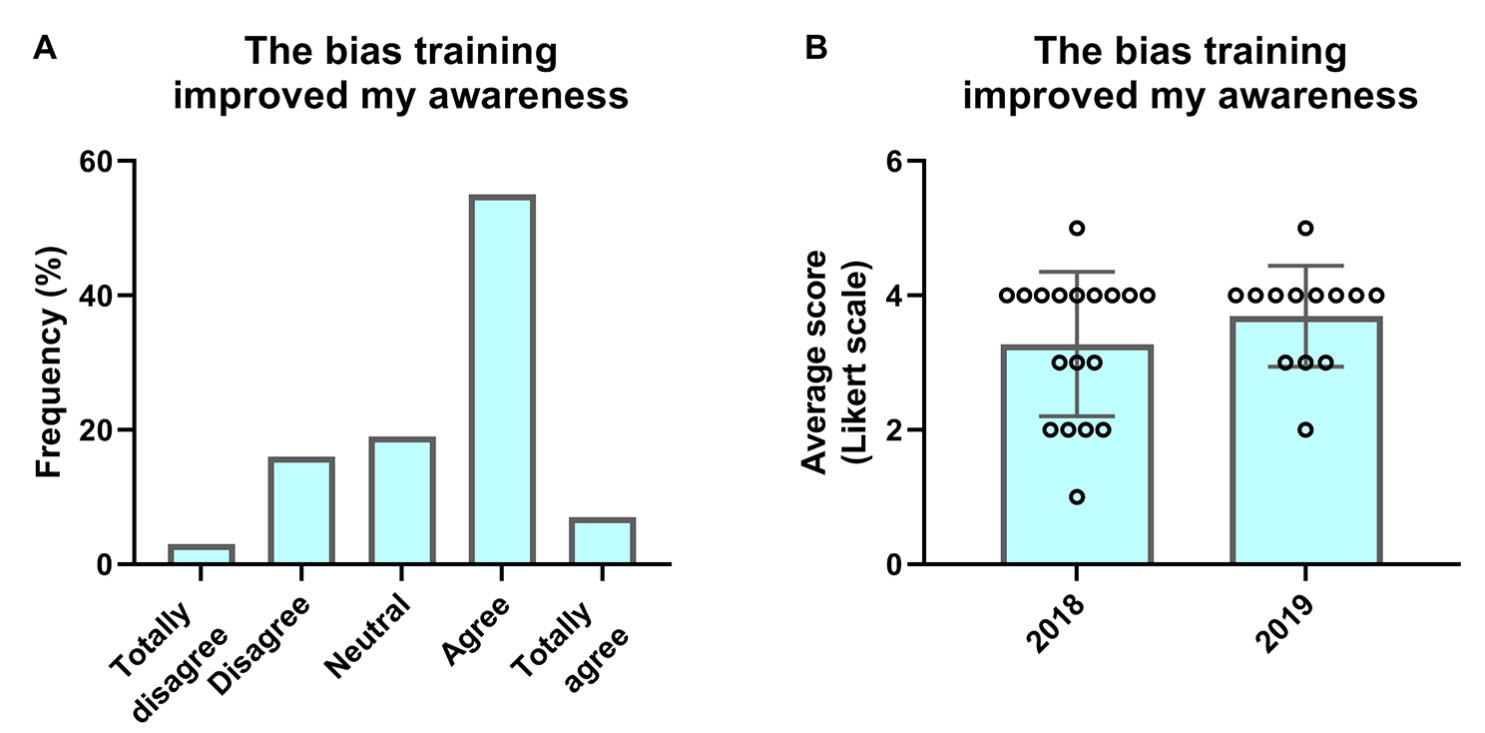
**Peer-mentor response to statement, “The bias-training helped increase my knowledge and awareness about D&I”**. (**A**) The mean results of 2018 and 2019 taken together, divided into the five categories of the Likert scale answer options. (**B**) The separate mean score for 2018 and 2019 (bar) and the individual answers (dots) for each year

The response rate to the mentor survey was 45% in the first year (n = 18) and 33% in the second year (n = 13), giving a total response rate of 39% (n = 31). In both years, the majority of the mentors indicated that the bias training had increased their awareness (Fig. 2). Figure 2a shows the results divided into the five categories of the Likert scale (from ‘totally disagree’ to ‘totally agree’). When combining the results from both years, 61% of the mentors stated they (totally) agreed with the statement, *“The bias training helped to increase my knowledge and awareness about diversity and inclusion,”* while 19% of the mentors were neutral and 20% stated they (totally) disagreed. Between 2018 and 2019, the number of mentors who agreed with this statement increased (Fig. 2b). Although the increase was not statistically significant, there was a positive trend, suggesting there was an improvement in the training and its effects between the two years.

**Fig. 3:**
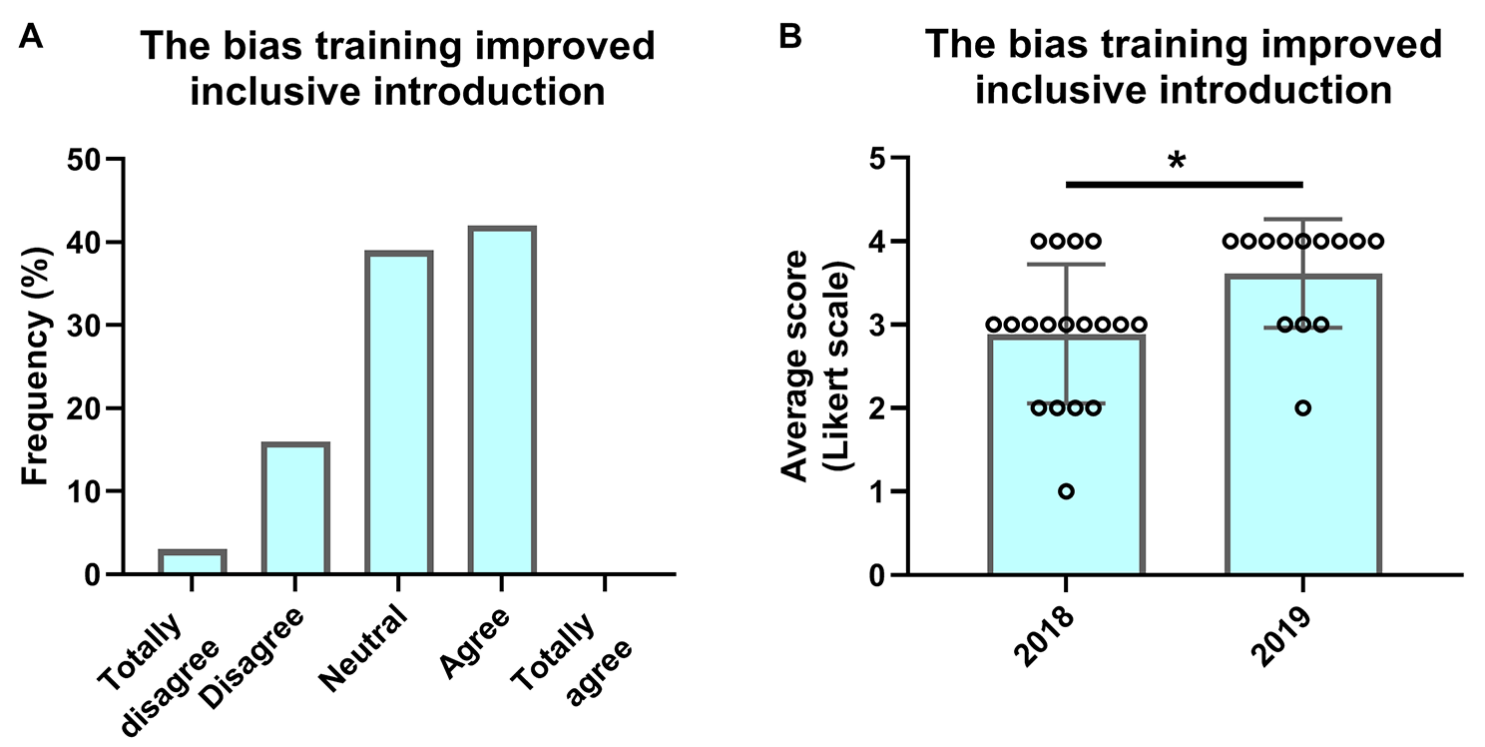
**Peer-mentor response to statement, “The bias-training helped me provide an inclusive introduction for first-year students”**. (**A**) The results of 2018 and 2019 taken together, divided into the five categories of the Likert scale answer options. (**B**) The separate mean score for 2018 and 2019 (bar) and the individual answers (dots) for each year. Statistical analysis shows a significant difference between 2018 and 2019 (Mann Whitney U test, W = 58,5, p = 0.014)

Figure 3 shows to what extent the bias training improved the mentors’ ability to create an inclusive orientation program. For both years, 42% of the mentors agreed with the statement, “*The bias training helped me provide an inclusive introduction for the first-year students*” (Fig. 3a). 19% of the mentors (totally) disagreed, whereas 39% of mentors remained neutral. Compared to 2018, there was a significant increase in the number of mentors who agreed with this statement compared to the year before (Fig. 3b, M = 2.89, *SD* = 0.83 in 2018 compared to *M* = 3.62, *SD =* 0.65 in 2019, *p =* 0.014), showing a significant improvement in the training and its effects between the two years.

Table 2 shows the qualitative data analysis of the explicit situations in which mentors were able to apply the knowledge gained during the bias training. The answers to the three knowledge application questions (Q15-17, appendix B) were analysed together, coded and placed into six categories: ‘general’, ‘age’, ‘diet’, ‘religion’, ‘alcohol’ and ‘other’ (ordered from least often to most often mentioned situations). The category ‘general’ refers to mentors who were unable to give specific examples but did feel they were generally more capable of creating an inclusive environment.

**Table 2:** How mentors could apply gained knowledge

Category	Number of mentions
Age	2
Diet	4
Religion	7
Alcohol	8
Other Inclusion	11
General	4
No Answer	34

Manners in which mentors were able to apply the knowledge gained from the bias training and increase inclusion of first-year students. Answers given by mentors were categorised based on common themes and topics within the answers

An example of a situation in ‘other’ was from a mentor who mentioned that one of the students in their group did not know how to ride a bicycle. The mentor wrote: “One of the students in my group couldn’t ride a bicycle very well. When going to the university we arranged for her to sit on the back of someone else’s bike so we could all stay together.” A second example was about a girl who had autism: “One of the girls in my group had autism. I was able to include her in most activities and in the end, she was really a part of the group.”

A quote from a mentor about a situation in the category ‘religion’ was: “We waited at the beginning of a meal to give those who wanted, a chance to pray.” The category ‘age’ mostly refers to the active inclusion of first-year students who were not yet 18, and the category ‘alcohol’ to situations where students were actively included despite the nature of the activity and the involvement of drinking alcohol. The mentor might have emphasised that participating in the beer chant could also be done with non-alcoholic beverages, for instance.

From the suggestions for improvements (categorised as ‘alcohol’, ‘communication’ and ‘diet’), we were able to distil different types of situations in which mentors thought students might feel excluded (Table 3). In both 2018 and 2019, alcohol remains the biggest issue, as there are still activities that revolve around the consumption of alcohol such as a beer chant, where students go to a pub and sing songs together (generally while drinking alcoholic beverages such as beer), or a pub crawl.

**Table 3:** Suggestions for improvements

Category	Number of mentions
Adaptations training	1
Diet	1
Communication	2
Alcohol	4
No Answer	52

Suggestions for improvements from mentors for the bias training or introduction program. Answers given by mentors were categorised based on common themes and topics within the answers

One mentor wrote about a situation concerning ‘diet’: “I noticed that vegetarians and people who eat Halal […] started getting the feeling that the organisation had not taken dietary wishes into account very well, or that it wasn’t taken seriously enough.”

The category ‘communication’ refers to the communication about activities toward students and how framing things differently can increase the inclusion of all students. One answer which falls in both the categories ‘alcohol’ and ‘communication’: “Change the name of the Gnome Drinks session to the La Chouffe Drinks session, because the word ‘gnome’ can be perceived as an insult by people.” [La Chouffe is a brand of beer which has a picture of a gnome on the label]. Although the number of suggestions was low, the issues are important and need to be addressed in the future.

### Internal consistency of the PGIS questionnaire

Although the PGIS questionnaire is a validated questionnaire, we determined the internal consistency of the questions in our sample, calculated by the Cronbach’s Alpha measure for the factors belonging and authenticity regarding mentors and peer students. The internal consistency was calculated with SPSS (IBM SPSS Statistics for Windows, version 26, IBM Corp., Armonk, N.Y., USA) and the resulting Cronbach’s Alpha was > 0.89 for all factors, indicating a high reliability between the sets of questions about belonging and authenticity. This was the case for both versions of the questionnaire.

### First-year students’ sense of inclusion during the orientation period

We also investigated the impact of the bias training on the perceived inclusion of first-year students. First-year students completed a survey based on the Perceived Group Inclusion Scale (PGIS; [14]). The response rate for 2018 was 19% (n = 91) and for 2019 it was 23% (n = 112). The total response rate was 21% (n = 203). We collected information on the background of the students (demographics) and on their perceived inclusion with respect to belonging and authenticity in relation to their fellow first-year students (peers) and in relation to their peer-mentor (Table 4). The results shown in Table 4 are the mean and standard deviation because differences are more visible as such.

**Table 4:** Results of student survey, including the (PGIS).

Characteristic	N (%)	Overall Inclusion	Belonging		Authenticity	
			Mentor	Peer students	Mentor	Peer students
**Year**						
2018	95 (46%)	3.90 (0.77)	3.98 (0.83)	3.97 (0.78)	3.82 (0.83)	3.84 (0.83)
2019	112 (54%)	4.08 (0.66)	4.16 (0.78)	4.04 (0.72)	4.14 (0.75)*	4.00 (0.73)
**Degree programme**						
Biomedical Sciences	109 (53%)	3.97 (0.79)	4.08 (0.87)	3.98 (0.83)	3.96 (0.85)	3.86 (0.86)
Medicine	97 (47%)	4.03 (0.63)	4.06 (0.73)	4.03 (0.65)	4.02 (0.74)	3.99 (0.68)
**Gender**						
Female	152 (74%)	3.95 (0.75)	4.02 (0.84)	3.96 (0.79)	3.94 (0.83)	3.88 (0.79)
Male	53 (26%)	4.14 (0.60)	4.23 (0.68)	4.13 (0.61)	4.14 (0.69)	4.04 (0.77)
**Disability**						
Yes	28 (14%)	3.96 (0.62)	4.11 (0.71)	3.93 (0.66)	4.01 (0.75)	3.77 (0.80)
No	178 (86%)	4.00 (0.73)	4.07 (0.82)	4.02 (0.76)	3.98 (0.81)	3.94 (0.78)
**Dyslexia**						
Yes	12 (6%)	4.02 (0.68)	4.12 (0.78)	4.03 (0.77)	3.94 (0.64)	3.97 (0.71)
No	194 (94%)	4.00 (0.72)	4.07 (0.81)	4.00 (0.75)	3.99 (0.81)	3.92 (0.79)
**Migration background**						
Yes	10 (5%)	4.09 (0.73)	3.98 (0.82)	3.91 (0.84)	4.31 (0.85)	4.16 (0.85)
No	197 (95%)	4.00 (0.72)	4.08 (0.81)	4.01 (0.74)	3.98 (0.80)	3.91 (0.78)
**Migration background parents**						
No	178 (86%)	4.01 (0.72)	4.08 (0.80)	4.03 (0.75)	3.98 (0.79)	3.95 (0.77)
One parent	17 (8%)	3.90 (0.77)	4.13 (0.93)	3.82 (0.73)	4.03 (0.96)	3.63 (0.94)
Both parents	12 (6%)	3.97 (0.61)	3.92 (0.80)	3.93 (0.68)	4.09 (0.79)	3.96 (0.73)
**First generation higher education?**						
No	159 (77%)	3.97 (0.75)	4.05 (0.84)	3.98 (0.78)	3.96 (0.84)	3.89 (0.81)
Yes	48 (23%)	4.11 (0.59)	4.18 (0.66)	4.11 (0.63)	4.09 (0.66)	4.03 (0.67)
**Sexual orientation**						
Heterosexual	186 (90%)	3.98 (0.73)	4.06 (0.82)	3.98 (0.76)	3.97 (0.81)	3.90 (0.79)
Homosexual	7 (3%)	4.29 (0.39)	4.39 (0.61)	4.39 (0.52)	4.31 (0.51)	4.09 (0.64)
Bisexual	14 (7%)	4.17 (0.58)	4.22 (0.71)	4.16 (0.55)	4.14 (0.74)	4.16 (0.65)
**Religion**						
Christian	50 (24%)	4.07 (0.66)	4.09 (0.67)	4.08 (0.70)	4.08 (0.72)	4.02 (0.77)
Islam	4 (2%)	3.98 (0.44)	3.78 (0.89)	4.00 (0.31)	4.19 (0.94)	3.94 (0.46)
No	152 (74%)	3.98 (0.74)	4.08 (0.85)	3.98 (0.77)	3.96 (0.83)	3.89 (0.79)

* Statistically significant difference (Mann Whitney U test, p = 0.001)

Data is shown as Mean (Standard Deviation) on a scale from 1 to 5. Overall scores for 2018 and 2019 are given at the top. Below, the data is shown per characteristic (degree programme, gender, disability, dyslexia, country of birth, parents’ country of birth, parents’ higher education, sexual orientation, and religion). Overall ‘Authenticity – Mentor’ in 2019 compared to 2018 is statistically significantly higher, indicated by * (Mann Whitney U test, p = 0.001)

In both years, the mean overall inclusion was high. In 2018 students scored the mean overall inclusion at 3.90 (SD = 0.77) and in 2019 at 4.08 (SD = 0.66) on a scale from 1 to 5. The differences in demographics did not measurably affect whether a student felt included (Table 4). Looking at each of the different aspects separately (degree programme, gender, disability, birthplace of student or parents, parents’ educational level, sexual orientation, and religion), the mean overall inclusion remained 3.90 or higher (SD = 0.77).

When analysing belonging and authenticity separately, both were given a high mean score by students. The results of the survey show that students felt that their peers made them feel that they belonged to the group (belonging, peer students) with a mean score of 3.97 (SD = 0.78) in 2018 and 4.04 (SD = 0.72) in 2019. The peer-mentor also made them feel they belonged (belonging, mentor) with a mean score of 3.98 (SD = 0.83) in 2018 and 4.16 (SD = 0.78) in 2019. Additionally, students made their peers feel they could be their authentic selves (authenticity, peer students) with a mean score of 3.84 (SD 0.83) in 2018 and 4.00 (SD = 0.73) in 2019, and the students were also made to feel they could be their authentic selves by their peer-mentors (authenticity, mentor) with a mean score of 3.82 (SD = 0.83) in 2018 and 4.14 (SD = 0.75) in 2019.

The quantitative data are supported by the student reports on the orientation activities and on their experiences with the mentors, which were categorised as ‘friendly and involved’, ‘inclusion during activities’, ‘approach’, ‘good atmosphere’ and ‘respect’ (Table 5).

**Table 5:** Student answers about effect of mentor on feeling of acceptance

Category	Number of mentions
Friendly and involved	59
Inclusion during activities	47
Approach	32
Good atmosphere	19
Respect	9
No answer	55

Student answers to the question, “How did the introduction committee and/or the mentor ensure that you felt accepted, valued, welcome, and at home during the introduction?” for 2018 and 2019. Answers were categorised based on common themes and topics within the answers

Results show that the students found the mentors consistently friendly and involved. One student wrote: “[The mentor] had a conversation with me. It made me feel seen as a person. She also tried to actively involve me in the student association, which made me feel she was happy I was there.” Moreover, most students said their mentor continually did their best to create cohesion in the group by including everyone and considering everyone’s opinion (category ‘inclusion during activities’). For example, a student said, “The mentor did his very best to ensure the group’s enthusiasm and to include everyone in everything. That was much appreciated and the atmosphere [in the group] was simply very pleasant.” Comments in the category ‘approach’ refer to mentors actively approaching students to ask how they experienced everything and how they were doing. One student wrote: “The mentor was very kind to us and very open. They really listened to everyone and gave the impression that they were genuinely interested. They also shared more personal stories of their own, making it easier for the rest of the group to share their stories.” Another student said: “Everyone was treated as an equal, [he] listened to everyone and tried to understand everyone.”

The students also valued the openness and respect the mentors showed, for instance when a student decided not to accompany the group to a party and their decision was respected (category ‘respect’). One student wrote: “When I decided not to go to bars, parties and such, the mentor said I was always welcome if I were to change my mind.” Another student said, “Nothing was obligatory, and your choices and opinions were appreciated.”

### Feeling of authenticity for first-year students in 2019

The data show that first-year students generally felt included in both 2018 and 2019. Additionally, the data showed an increase in the mean score for authenticity that the students experienced due to their mentor (mean score of 3.82 (SD = 0.83) in 2018 and 4.14 (SD = 0.75) in 2019). Statistical analysis showed this difference was statistically significant (Mann-Whitney U test, W = 3977, p = 0.001; see Table 4). One of the underlying reasons could be the improvements made to the training between 2018 and 2019. Mentors found the training more useful in 2019, with the number of mentors agreeing with this statement increasing from 17 to 30% between the two years (data not shown). The increased level of authenticity the students felt, support the previously presented data showing the mentors felt better able to provide an inclusive introduction in 2019 compared to 2018 (Fig. 2b).

Although students were pleased with the atmosphere and the inclusion, there were still aspects of the introduction which require further attention. The answers to the question, “What can the introduction committee and/or the mentor do to make you feel more accepted, valued, welcome and at home?” were categorised as ‘alcohol and party alternatives’, ‘increased interaction’, ‘organisation’, ‘increased inclusion’ and ‘other’ (Table 6).

**Table 6:** Student answers to about what could help to increase feeling of acceptance

Category	Number of mentions
Other	6
Increased inclusion	7
Organisation	11
Increased interaction	11
Alcohol and party alternatives	16
No answer	157

Student answers to the question, “What can the introduction committee and/or the mentor do to make you feel more accepted, valued, welcome and at home?” for 2018 and 2019. Answers were categorised based on common themes and topics within the answers

Although most students did not have a suggestion for improvements, there were still some comments to consider for the future. Of the suggestions that were given, most fell into the category ‘alcohol and party alternatives’, where students mentioned that there were not enough alternatives for the people who did not (want to) drink alcohol. One student wrote: “The orientation programme was very much about partying and alcohol in my opinion. Maybe an alternative evening programme for people who aren’t interested in partying would help. The programme could’ve also contained a cool assignment about the university.” Another student agreed with their answer: “If there is a party in the evening and I didn’t want to go, there wasn’t an alternative. Maybe they could take that more into account, that not everyone likes to party.” This is an important issue which needs to be managed better in future programmes.

An example of an improvement from the ‘organisation’ category was that more ‘free’ time should be scheduled for people to unwind and relax on their own. The schedule was quite full and for people who are more introverted, this can be tiring, as supported by one student’s answer: “I am quite introverted and I need time to recover after, for example, a big party. During the orientation programme I got the feeling that the programme was directed more at extroverts. If the amount of time between a party and the next activity would just be a bit longer, it would have helped me a lot. Now I often had to go home early because I get tired quickly during parties.”

Some students would have liked more activities to get to know each other better, categorised as ‘increased interaction’. One student wrote: “Even more activities or games that are specifically aimed at getting to know each other and creating a group feeling.” Another wrote: “More interaction with other groups, now I only got to know my own group really well.” One student also mentioned: “Maybe make sure that the groups are encouraged to come to all the orientation activities.”

## Discussion

Previous studies on diversity have shown that a diverse population can present people with challenges [7, 29], but can also have beneficial effects for students in HE [5–8, 48]. It is important for all students to feel included [7, 14]. One way to create an inclusive environment is by increasing self-awareness of implicit bias [9]. To work towards an inclusive academic orientation programme, we developed and provided a bias training for peer-mentors who guide new Biomedical and Medical students in their transition to HE. The training included both theoretical and practical components on diversity and inclusion to help create an increased awareness of unconscious bias. The effects of the training were then studied on two levels: (1) the perceived inclusion of the first-year students, and (2) the awareness and behavioural changes in the peer-mentors. There have been several studies regarding the positive impact of mentoring [49] and, more specifically, the use of peer-mentors for student adjustment during their transition to HE [25–27, 50]. However, we found that there are only a limited number of studies that focused on the use of peer-mentors to increase students’ sense of belonging at university [27]. Therefore, the focus of this study was to see whether peer-mentors helped to create an inclusive environment during the orientation programme for first-year students to assist their transition to HE.

Overall, the peer-mentors that participated in this study indicated that the bias training increased their awareness about diversity, inclusion, and unconscious bias. The improvements made in the training between the first and second year of the study resulted in peer-mentors who felt significantly more able to provide an inclusive orientation compared to the previous year. Generally, the peer-mentors were pleased with the training, with one mentor commenting, “Thank you for the training, I learned valuable lessons which I will be able to use for the rest of my life!”

While many of the studies conducted on students’ sense of belonging and their transition to HE focused on interventions directed at incoming students (e.g., focus on participants’ awareness of own background and identity and how that influences their perception of situations) [51–53], this study, focused on an intervention directed at the institutional side (e.g., training and using peer-mentors). According to Wilcox et al. (2006), one of the important factors for student persistence in HE is social support [54]. This includes being able to form friendships with other students. Peer-mentors can play an important role in this process, as they can guide first-year students and influence community building within the orientation group [26, 27]. Therefore, it is important to pay attention to both sides (institution and incoming student) to ensure the interventions can complement and reinforce each other and build a stronger sense of belonging in incoming students.

Triangulating the quantitative and qualitative results of this study, first-year students, overall, experienced the orientation programme as inclusive, and they felt they belonged to the group while remaining their authentic selves. This result correlates to the findings for the peer-mentors, who felt their behaviour during the orientation period was more inclusive due to their training. These findings are in line with previous studies on peer-mentorship which has shown that peer-mentors can contribute to a smooth transition into HE [24–27].

When further analysing the quantitative student data, we found no correlation between the different demographic categories of our respondents (place of birth, parents’ education, religion, sexual preference, etc.) and their experienced level of inclusion. It is possible that belonging to a group where there is a common interest, belief, or preference, such as religion or sexual preference, can also result in a high level of inclusion within their in-group, even if the in-group is a minority. Furthermore, the students may experience that they are part of the same in-group due to having chosen the same degree program. Speculatively, this may explain the lack of correlation in this study between level of inclusion and demographic category.

Although the level of inclusion was high throughout the orientation programme, the students and mentors had some critical remarks in relation to the activities. Both students and mentors indicated that the consumption of alcohol remains a large obstacle to inclusion during social activities. A few activities such as pub-crawls and beer-chants are a regular part of the schedule. Although peer-mentors try to emphasize that these activities do not have to involve alcohol consumption, the first-year students are left with limited choice, leaving many to feeling pressured to drink alcohol. It is important to change the perception of these activities or to offer sufficient alternatives to foster inclusion for all students.

### Limitations

One of the limitations of this study is that the effect measured in mentors was self-reported. The mentors received a survey with questions about the usefulness of the training and whether it helped them to create an inclusive atmosphere during the introduction. It is possible that the information was affected by recall period, social desirability, or selective recall. In order to counteract these possible effects, we measured the perceived inclusion of the first-year students using the validated PGIS [14]. As stated by Stes et al. (2010): “students’ perceptions of the teaching and learning environment encompass their perception of teachers’ behaviour.” [55] Using both these measures and combining quantitative and qualitative data, we ensured that we could measure the change in behaviour of the peer-mentors who followed the training session in a reliable manner.

A second potential limitation of this study was the absence of a control group. The perceived inclusion of the first-year students was not measured in a cohort where the peer-mentors were not trained beforehand. As the peer-mentorship was a pre-existing part of the orientation programme, it seemed more ethical to train all peer-mentors and not just a portion of them. The perceived inclusion we measured in first-year students in this study was generally high. Compared to the reports previously given by our exploratory focus groups, which gave us cause to begin this intervention, the overall high feeling of inclusion is a strong indication of a positive effect of the bias training.

Another limitation is that we also only observe self-reported changes in behaviour after a single workshop. It has previously been suggested that one-time events have a lower impact than instructional development over a longer period of time [55]. Consequently, it is important that this training be reinforced by follow-up training sessions and other activities regarding inclusion of students.

A final limitation was the sample size for the peer-mentor portion of the study. Of the 80 possible participants, only 31 mentors participated in the study. A future study to specifically investigate the effect of bias awareness training on, for instance, change in behaviour, should include a larger sample size to draw sound conclusions.

### Future perspectives

As already mentioned, peer-mentors can ensure a smooth transition to HE [25–27, 50] and we have shown that they are able to create an inclusive atmosphere during the orientation period. To ensure continuity, it is necessary to offer the awareness training on a yearly basis. Continuous improvement and evaluation of the bias training and the activities during the orientation period will enhance the perception of belonging and authenticity for every student during the transition to HE. We encourage other institutions of HE to learn from this approach by providing bias awareness trainings for their (peer-)mentors to enhance inclusive orientation periods for every student. In addition, we will investigate the perceived inclusion among students beyond the orientation period during their entire education, as a sense of belonging is often challenged, for example during internships. Additionally, the impact of the perceived inclusion on students’ academic performance will be investigated.

Offering additional training sessions is important to ensure lasting behavioural change [56–58]. It is important to ensure an inclusive curriculum where the presence of stereotypes and other reinforcements of implicit bias are minimized [59, 60]. We chose to start training peer-mentors because they are the bridge between first-year students and faculty during their transition to HE. To provide an inclusive academic environment for students throughout the entire degree programme, we have initiated training sessions for teachers and other academic staff. The effects of these interventions will also be researched. It is necessary that students not only feel they belong to the group during the transition into HE, but that they can be themselves and feel accepted as they are during their entire academic career.

Generally, the cohorts studied in this investigation were not highly diverse and we found no correlation between the different demographic categories of our respondents and their experienced level of inclusion. Further research would be required to investigate whether or not the diversity in this group was, in fact, low. It is possible that the few students from a minority background were students from more privileged environments who do not feel exclusion due to their cultural or religious background. A last possibility is that there are aspects of diversity which we did not include in our questionnaire and were therefore not investigated.

Although one might expect that there is no immediate reason to study inclusion in a homogenous population, it remains important as every person is an individual with their own unique background and interests. It has been shown that inclusive education in a diverse classroom prepares students for societal challenges [9–13]. Working to increase the diversity is also an issue we must pay attention to, as being part of a diverse team improves team and study performances [5, 7, 8]. This has also specifically been shown in scientific settings, where scientific and medical teams benefit from diversity [6, 8, 61]. In medical schools, it is important to equip students with the tools to successfully treat a diverse population of patients. With diversity in the student body, students will be able to learn from each other and benefit from observing interactions between their classmates with a different background and a patient who might share that background [62].

When it comes to perceived inclusion, it is important to address multiple levels within the organization at once, since each level influences the others. At the School of Medicine of Utrecht University, we have initiated interventions which address the numbers (e.g., outreach to secondary schools and minority groups to enrol in our academic programmes), address the knowledge (e.g., researching the effects of bias awareness trainings), and address the institution (e.g., changing the culture within the organization to be more inclusive) [63]. We are working towards Diversity 3.0, where “diversity and inclusion are integrated into the core workings of the institution and [are both] integral to achieving excellence,” [63, 64]. It is important to target the students individually by increasing their awareness of their own implicit bias, but it is equally important to change the culture at an institutional level [9, 65–67]. This change should be apparent from the start of academic education.

The model that we present here is one that could be easily implemented at other degree programmes or educational institutions, where senior students form a bridge between the first-year student and faculty by offering peer-mentoring to the first-year students. This is already happening, as other schools of our university are using the knowledge we have gained and shared to start their own awareness training sessions and have asked us to train their peer-mentors and faculty members. Additionally, the university has asked us to give multiple training sessions to students in student-government positions and other institutions in the Netherlands have asked for the knowledge we have gained in this study to implement at their own institutions. Overall, we conclude that training peer-mentors is an effective way to increase awareness and contributes to an inclusive atmosphere during the start of higher education.

## Conclusion

Taken together, the bias training was experienced as useful by mentors, having improved their awareness, and enabling them to provide an inclusive orientation. Overall, students felt a high degree of inclusion, both in belonging and authenticity. The changes made to the bias training between the first and second year improved the training, as shown by the increased number of mentors who found they were able to provide an inclusive introduction in year two and by the significantly higher number of students whose mentor made them feel they could be their authentic selves. Overall, we conclude that training peer-mentors is an effective way to ensure an inclusive atmosphere and a smoother transition for first-years students to HE.

### Electronic supplementary material

Below is the link to the electronic supplementary material.

Supplementary Material 1

## Data Availability

The datasets generated and/or analysed during the current study are not publicly available due to the personal nature of the data but are available from the corresponding author on reasonable request.

## List of Abbreviations

HE: Higher education
STEM: Science, technology, engineering, and mathematics
UMC Utrecht: University Medical Centre Utrecht
PGIS: Perceived group inclusion scale
