# Supplementary material for: Peer-mentorship and first-year inclusion: building belonging in higher education
Source: BMC Med Educ. 2023 Nov 7;23:833. doi: 10.1186/s12909-023-04805-0 (PMC10629167; doi:10.1186/s12909-023-04805-0)
Supplement: Supplementary file 1 — Supplementary Material 1 [file 12909_2023_4805_MOESM1_ESM.docx]

# Appendix

## Appendix A: Questions Peer-Mentor Survey (2018 + 2019)

Q 1: In which year did you start your degree programme?

Q 2: How many times have you been a mentor?

Q 3: Which degree programme do you follow at the moment?

Q 4: Which gender do you identify with?

Q 5: Do you have a professionally diagnosed disability, either mental or physical, with regard to learning or otherwise? (e.g., handicap, dyslexia, etc.)

Q 6: In which country were you born?

Q 7: In which country were your parent born?

Q 8: Has at least one of your parents followed higher education?

Q 9: What is your sexual orientation?

Q 10: Are you religious and if so, what is your religion?

Q 11: The bias training on 25/08/2019 increased my knowledge and awareness of diversity and inclusion.

Q 12: Can you elaborate on your answer?

Q 13: The bias training on 25/08/2019 contributed to my ability to organise an inclusive introduction for the first year students.

Q 14: Can you elaborate on your answer?

Q 15: Can you think of a situation or an activity during which you were able to implement the knowledge you gained from the bias training?

Q 16: Can you think of a situation or an activity during which, due to the bias training, you made sure that specific students would feel accepted, welcome and at home?

Q 17: Can you think of a situation or an activity during which, due to the bias training, you noticed that someone did not feel accepted, welcome or at home?

Q 18: Can you think of an activity from the introduction programme which you would like to adapt or omit from the programme for next year because it was not inclusive for all students?

Q 19: Do you have any additional comments or questions?

## Appendix B. Questions Student Survey (2018)

Q 1: Which degree programme do you follow at the moment?

Q 2: Which gender do you identify with?

Q 3: Do you have a professionally diagnosed disability, either mental or physical, with regard to learning or otherwise? (e.g., handicap, dyslexia, etc.)

Q 4: In which country were you born?

Q 5: In which country were your parent born?

Q 6: Has at least one of your parents followed higher education?

Q7: What is your sexual orientation?

Q 8: Are you religious and if so, what is your religion?

### PGIS

During the introduction, the group of my fellow students…

Q 9: …gives me the feeling I belong to the group.

Q 10: …gives me the feeling I am part of the group.

Q 11: …gives me the feeling I fit in the group.

Q 12: …treats me like an insider.

Q 13: …likes me.

Q 14: …values me.

Q 15: …is happy with me.

Q 16: …cares for me.

Q 17: …allows me to be authentic.

Q 18: …allows me to be who I am.

Q 19: …allows me to express my authentic self.

Q 20: …allows me to show myself as I am.

Q 21: …encourages me to be authentic.

Q 22: …encourages me to be who I am.

Q 23: …encourages me to express my authentic self.

Q 24: …encourages me to show myself as I am.

During the introduction, the mentor…

Q 25: …gives me the feeling I belong to the group.

Q 26: …gives me the feeling I am part of the group.

Q 27: …gives me the feeling I fit in the group.

Q 28: …treats me like an insider.

Q 29: …likes me.

Q 30: …values me.

Q 31: …is happy with me.

Q 32: …cares for me.

Q 33: …allows me to be authentic.

Q 34: …allows me to be who I am.

Q 35: …allows me to express my authentic self.

Q 36: …allows me to show myself as I am.

Q 37: …encourages me to be authentic.

Q 38: …encourages me to be who I am.

Q 39: …encourages me to express my authentic self.

Q 40: …encourages me to show myself as I am.

### Open-ended questions:

Q41: In which way did the orientation committee and/or the mentor make sure you felt accepted, appreciated, and welcome or contributed to a high sense of belonging during the orientation period?

Q42: Which improvements can the orientation committee and/or mentor make during the orientation period to make sure you feel accepted, appreciated, and welcome or to contribute to a higher sense of belonging?

Q43: You can leave any additional comments here.

## Appendix C: PGIS-Questions Student Survey (2019) (the first 8 questions were the same as 2018)

During the introduction, the group of my fellow students…

Q 9: …gives me the feeling I belong to the group.

Q 10: …allows me to be who I am.

Q 11: …treats me like an insider.

Q 12: …allows me to express my authentic self.

Q 13: …values me.

Q 14: …encourages me to be who I am.

Q 15: …cares for me.

Q 16: …encourages me to express my authentic self.

During the introduction, the mentor…

Q 17: …gives me the feeling I belong to the group.

Q 18: …allows me to be who I am.

Q 19: …treats me like an insider.

Q 20: …allows me to express my authentic self.

Q 21: …values me.

Q 22: …encourages me to be who I am.

Q 23: …cares for me.

Q24: …encourages me to express my authentic self.

### Open-ended questions:

Q25: In which way did the orientation committee and/or the mentor make sure you felt accepted, appreciated, and welcome or contributed to a high sense of belonging during the orientation period?

Q26: Which improvements can the orientation committee and/or mentor make during the orientation period to make sure you feel accepted, appreciated, and welcome or to contribute to a higher sense of belonging?

Q27: You can leave any additional comments here.

### Appendix D: Content of the bias awareness training

Before the start of the orientation programme, peer-mentors received two days of trainings to help prepare them for their mentorship tasks. A bias awareness training based on the six-point framework was added to the curriculum.

The training started by providing an overview of the course and its learning goal: to become aware of (your own) implicit biases and how they may influence (your) behaviour. Ground rules were explained, and the participants were reassured that everything discussed during the course would remain private. We also explicitly requested the participants remain respectful of each other to help ensure an open and sharing environment. Differences of opinion were allowed if they were discussed respectfully (framework point 1).

After the ground rules were established, we then continued with an exercise called *Over the Line*. In this exercise, participants lined up next to each other. Different statements were read aloud and if applicable to a participant, they would cross over to the other side of the line. Example statements included: ‘I was the first in my family to study at a higher education institution’, ‘I know a fellow student from a minority background’ and ‘When growing up I always had people around me who could advise me on my future or my education’. These statements aimed to increase awareness of different forms of diversity and to highlight that not all forms of inequality are visible. This exercise also helps raise awareness of participant’s own implicit biases (framework point 4).

After this exercise, the peer-mentors were provided with background theory regarding unconscious or implicit bias, diversity and inclusion and their benefits within a team, within education, and within science in general. Different examples were given where implicit bias had had a negative outcome and where diverse perspectives were lacking, such as the case when self-driving cars were first developed and did not recognize dark-skinned people as well as they recognized light-skinned people (45). Examples of bias in medicine were also shared, such as the differences in cardiovascular disease between males and females (46). These examples were used to highlight the importance of bias awareness and to show the positive effects of diversity (framework points 3 and 6). Following this exercise, we then explained the theory behind mental heuristics and the reason our brains categorize and associate information we receive with knowledge we already have. We also discussed a few commonly occurring cognitive biases in more detail such as halo effect (the tendency for positive impressions of a person, in one area to positively influence one's opinion or feelings in other areas) or confirmation bias (the tendency to search for, interpret, favor, and recall information in a way that confirms or supports one's prior beliefs or values) (47) (framework point 2).

In the next session of the training, participants were divided into groups of four and five people and were asked to think of activities during orientation where implicit biases could lead to exclusion of first-year students. They were then asked to think of alternative activities to replace these ‘problem’ areas (framework points 3, 5 and 6). After the small group discussions, the groups came together for a plenary discussion where they addressed the problem areas within the orientation programme and potential solutions. The groups provided feedback to each other, and the larger group discussion led to further improvements and solutions for the orientation programme (framework points 4, 5 and 6). We finished the training by summarizing the points discussed. Our closing take-home message for the peer-mentors was that everyone has biases, but by becoming aware of your own unconscious biases, you can prevent them from influencing your behaviour.

### Changes made to the second year

After the first-year training weekend, participants commented in the session evaluation that they found the theoretical component of the training long and as a result not sufficiently engaging. As a response to this feedback, in the second year of our study, we shortened the theoretical component, keeping the set-up of the training as described above but with fewer examples of instances where diversity was lacking and of where diversity offered benefits. We also chose to leave out some of the forms of cognitive bias which were less relevant to the first-year orientation programme. See figure 1 in the main body of the article.
